# Identification of a novel senolytic agent, navitoclax, targeting the Bcl‐2 family of anti‐apoptotic factors

**DOI:** 10.1111/acel.12445

**Published:** 2016-03-18

**Authors:** Yi Zhu, Tamara Tchkonia, Heike Fuhrmann‐Stroissnigg, Haiming M. Dai, Yuanyuan Y. Ling, Michael B. Stout, Tamar Pirtskhalava, Nino Giorgadze, Kurt O. Johnson, Cory B. Giles, Jonathan D. Wren, Laura J. Niedernhofer, Paul D. Robbins, James L. Kirkland

**Affiliations:** ^1^ Robert and Arlene Kogod Center on AgingMayo ClinicRochesterMNUSA; ^2^ Department of Metabolism and AgingThe Scripps Research InstituteJupiterFLUSA; ^3^ Center of Medical Physics and TechnologyHefei Institutes of Physical SciencesHefeiChina; ^4^ Arthritis and Clinical Immunology Research ProgramOklahoma Medical Research FoundationOklahoma CityOKUSA

**Keywords:** ABT‐263, Bcl‐2 family, dasatinib, quercetin, senescent cells, TW‐37

## Abstract

Clearing senescent cells extends healthspan in mice. Using a hypothesis‐driven bioinformatics‐based approach, we recently identified pro‐survival pathways in human senescent cells that contribute to their resistance to apoptosis. This led to identification of dasatinib (D) and quercetin (Q) as senolytics, agents that target some of these pathways and induce apoptosis preferentially in senescent cells. Among other pro‐survival regulators identified was Bcl‐xl. Here, we tested whether the Bcl‐2 family inhibitors, navitoclax (N) and TW‐37 (T), are senolytic. Like D and Q, N is senolytic in some, but not all types of senescent cells: N reduced viability of senescent human umbilical vein epithelial cells (HUVECs), IMR90 human lung fibroblasts, and murine embryonic fibroblasts (MEFs), but not human primary preadipocytes, consistent with our previous finding that Bcl‐xl siRNA is senolytic in HUVECs, but not preadipocytes. In contrast, T had little senolytic activity. N targets Bcl‐2, Bcl‐xl, and Bcl‐w, while T targets Bcl‐2, Bcl‐xl, and Mcl‐1. The combination of Bcl‐2, Bcl‐xl, and Bcl‐w siRNAs was senolytic in HUVECs and IMR90 cells, while combination of Bcl‐2, Bcl‐xl, and Mcl‐1 siRNAs was not. Susceptibility to N correlated with patterns of Bcl‐2 family member proteins in different types of human senescent cells, as has been found in predicting response of cancers to N. Thus, N is senolytic and acts in a potentially predictable cell type‐restricted manner. The hypothesis‐driven, bioinformatics‐based approach we used to discover that dasatinib (D) and quercetin (Q) are senolytic can be extended to increase the repertoire of senolytic drugs, including additional cell type‐specific senolytic agents.

## Introduction

Senescent cells contribute to age‐related diseases (Zhu *et al*., 2014). Much like cancer cells, senescent cells are resistant to apoptosis (Wang, 1995; Fridman & Lowe, 2003), potentially protecting them from their own pro‐inflammatory secretions, reactive metabolites, and activated DNA damage response. They are instead eliminated by the immune system (Xue *et al*., 2007). We therefore hypothesized that senescent cells depend upon anti‐apoptotic defenses similarly to cancer cells. Indeed, our analysis of the transcriptome of senescent human preadipocytes identified pro‐survival pathway upregulation (Zhu *et al*., 2015).

**Figure 1:**
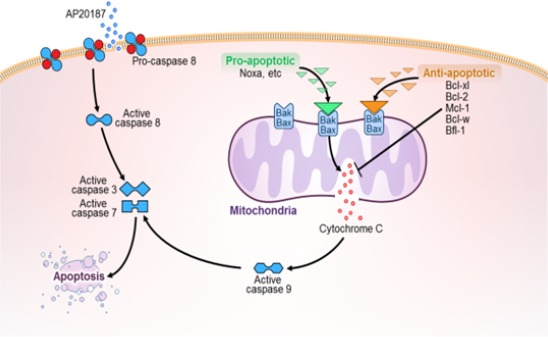
Apoptotic pathways. Bcl‐2 family members act upstream of mitochondrial‐mediated apoptosis. Pro‐apoptotic BH3‐only proteins (e.g., Noxa) transiently bind Bax/Bak, facilitating activation, while anti‐apoptotic Bcl‐2 family members (e.g., Bcl‐xl, Bcl‐2, Mcl‐1, Bcl‐w) block Bax/Bak directly. AP20187, which activates the caspase‐8 moiety of the myristoylated membrane‐bound ATTAC fusion protein product of the *INK‐ATTAC* transgene, is also shown. Both the caspase‐8‐ and Bak/Bax/cytochrome c‐related pathways activate the executioner caspases 3 and 7.

To eliminate senescent cells, we devised the strategy of using a senescence‐activated promoter to drive *ATTAC*, which encodes a caspase‐8 moiety inducible by the drug AP20187. Caspase‐8 activates the caspase‐3/7 executioner cascade (Fig. 1). AP20187 eliminated senescent cells and alleviated age‐related dysfunction in progeroid mice producing ATTAC only in p16^Ink4a^‐expressing cells (Baker *et al*., 2011). Furthermore, we recently demonstrated that AP20187 clears senescent cells from naturally aged 18‐month‐old INK‐ATTAC mice and enhances fat tissue function (Xu *et al*., 2015). These findings indicate that senescent cells can be removed by disabling pro‐survival pathways upstream of caspases 3 and 7. Indeed, based in the hypothesis that targeting anti‐apoptotic, pro‐survival pathways is a means for eliminating senescent cells selectively, we found that the combination of D and Q induces apoptosis of particular types of senescent cells and that the combination alleviates senescence‐related phenotypes in mice (Zhu *et al*., 2015). We also reported that siRNA targeting Bcl‐xl, a member of the anti‐apoptotic Bcl‐2 family, reduced viability of senescent but not nonsenescent HUVECs, while having little effect on senescent or nonsenescent human preadipocytes. This result implicated the members of the anti‐apoptotic Bcl‐2 family as an Achilles heel of at least certain types of senescent cells (Zhu *et al*., 2015). Here, we confirm that targeting Bcl‐2 proteins is a senolytic strategy by demonstrating that the drug, navitoclax (N; ABT‐263), which targets several Bcl‐2 family proteins, is senolytic in HUVECs and IMR90 cells, but not human primary preadipocytes. Furthermore, while this paper was under review, the senolytic effect of targeting Bcl‐2 family members was confirmed by others using an RNA interference approach analogous to the one first published and with N (Chang *et al*., 2015).

Like D, N and T are used to induce apoptosis of cancer cells. N is used to treat lymphoid malignancies, small‐cell lung cancer, and chronic lymphocytic leukemia (Wendt, 2008; Vogler *et al*., 2009; Billard, 2013). T is being investigated for treating certain types of cancer (Wang *et al*., 2006). Also like D and Q, N and T have multiple targets. N targets Bcl‐xl, Bcl‐2, and Bcl‐w, and T targets Bcl‐xl, Bcl‐2, and Mcl‐1 (Vogler *et al*., 2009). These Bcl‐2 proteins function to prevent apoptosis through safeguarding mitochondrial outer membrane integrity by binding to Bax/Bak and disrupting interactions among Bcl‐2 family proteins upstream of the executioner caspases (see Fig. 1 (Kang & Reynolds, 2009; Vogler *et al*., 2009; Strasser *et al*., 2011; Czabotar *et al*., 2014; Correia *et al*., 2015)).

Like D and Q, N is not universally senolytic and acts in a senescent cell type‐dependent manner. N is senolytic in HUVECs, IMR90 cells, and MEFs, but not in senescent human primary preadipocytes. Based on these senescent cell type‐specific effects of N and its ability to treat cancers with specific Bcl‐2 family signatures (Tahir *et al*., 2010; Vo *et al*., 2012), we also tested signatures of Bcl‐2 family member proteins and found these signatures do indeed correlate with susceptibilities of different senescent cell types to N. This suggests that the molecular signatures of different types of senescent cells may be of utility in predicting responsiveness to Bcl‐2 family inhibitors or potentially to other classes of senolytic drugs.

## Results

### Effects of Bcl‐2 inhibitors on senescent cells

**Figure 2:**
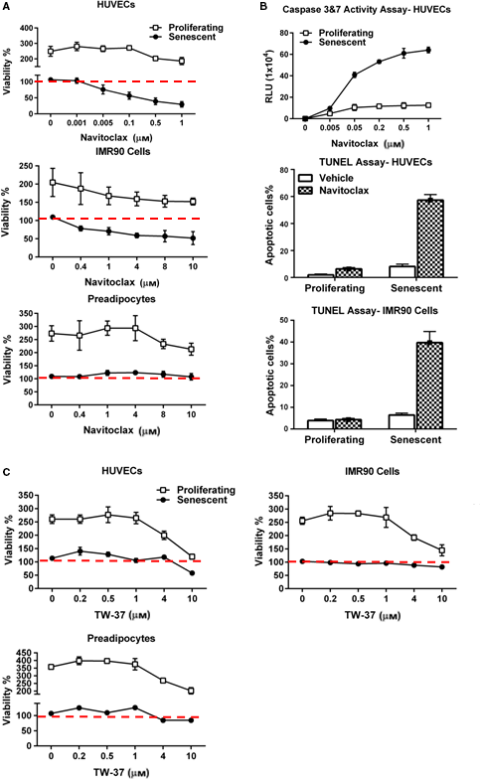
N targets senescent cells. (A) N is more effective in reducing viability (ATPLite) of senescent HUVECs and IMR90 cells than human preadipocytes. Proliferating or senescent cells were exposed to different concentrations of N for 3 days. The red line denotes plating densities at day 0 of senescent and nonsenescent cells, both set to 100%. HUVEC and IMR90 data represent means ± SEM of four replicates at each drug concentration. Preadipocyte data are means ± SEM of four different subjects; *P *<* *0.001, ANOVA. (B) N induces apoptosis in senescent HUVECs and IMR90 cells. HUVECs were treated with N for 12 h, and then caspases 3 and 7 were assayed using a luminescent substrate. N (1000 nM) induced apoptosis in senescent cells by TUNEL assay; *P *<* *0.001, *t*‐test. (C) The Bcl‐2/Bcl‐xl inhibitor, TW‐37, does not impact the viability of senescent HUVECs, IMR90 cells, or human preadipocytes. Cells were exposed to different concentrations of TW‐37 for 3 days. The red line denotes plating densities on day 0 of senescent (set to 100%) and nonsenescent cells (also set to 100%). HUVEC and IMR90 cell data are means ± SEM of four replicates at each concentration. Preadipocyte data are means ± SEM of four different subjects.

**Figure 3:**
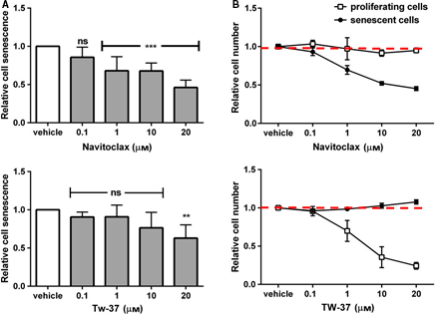
Navitoclax selectively kills senescent *Ercc1*‐deficient murine embryonic fibroblasts, while TW‐37 is less senolytic. Cultures containing ~ 50% senescent MEFs were exposed to drugs for 48 h prior to analysis of SA‐βGal^+^ cells using C_12_ FDG and Hoechst staining to determine (A) the ratios of senescent to nonsenescent cells within each of the wells as a function this ratio in vehicle‐treated cells. This indicates that T only eliminates senescent cells at high concentrations. (B) We also determined the ratio of the number of senescent (or proliferating) cells in each well to the number of senescent (or proliferating) cells in the corresponding vehicle‐treated wells (red dotted lines). This indicates that T actually reduces proliferating cell numbers at concentrations below those that reduce senescent cells. The data shown are means ± SD of 3 replicates; **P <* *0.01; ***P <* *0.001, ANOVA.

N selectively induced apoptosis in radiation‐induced senescent HUVECs and IMR90 cells (Fig. 2A,B). Cell death by apoptosis was confirmed by caspase‐3&7 and TUNEL assays (Figs 2B and S1). N did not induce apoptosis of senescent human preadipocytes or proliferating, nonsenescent HUVECs or IMR90 cells. T was less senolytic than N in HUVECs and IMR90 cells and was not senolytic in primary human preadipocytes (Fig. 2C). We also tested N and T in cultures containing a mixture of senescent and nonsenescent *Ercc1*‐deficient MEFs induced to undergo senescence by passaging and oxidative stress, as opposed to radiation (Fig. 3). Using an assay based on counting surviving senescence‐associated β‐galactosidase (SA‐βGal)‐positive vs. β‐galactosidase‐negative cells after adding drugs to these cultures, we found that N was senolytic while T was not. Indeed, T induced apoptosis of proliferating, but not senescent MEFs.

### Bcl‐2 family member siRNA analyses in senescent vs. nonsenescent cells

**Figure 4:**
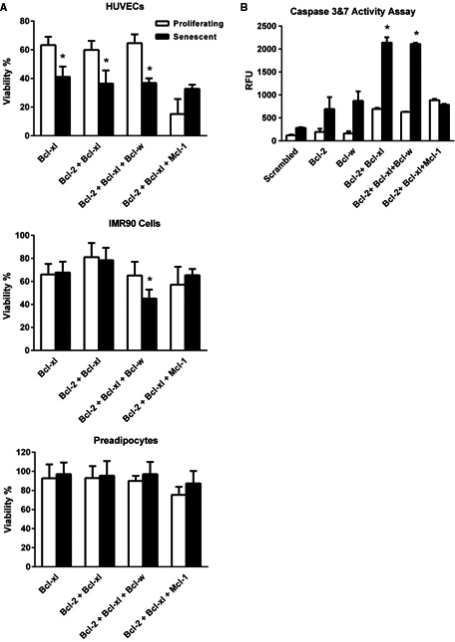
Senolytic effects of targeting Bcl‐2 family members by RNA interference. (A) Proliferating or senescent HUVECs, IMR90 cells, or primary human preadipocytes were transfected with siRNAs individually or in combination 2 days before viability analysis (ATPLite). Decreases in targeted mRNAs were confirmed by RT–PCR (Fig. S2). Bcl‐xl siRNA alone or in combination with Bcl‐2 and Bcl‐xl siRNA was senolytic in HUVECs, but not IMR90 cells or preadipocytes. The combination of Bcl‐2, Bcl‐xl, and Bcl‐w siRNAs reflects the targets of N, while Bcl‐2, Bcl‐xl, and Mcl‐1 reflects those of T. HUVEC and IMR90 cell data are means ± SEM of five replicates at each concentration. Preadipocyte data are means ± SEM of four different subjects. *P <* *0.05, *t*‐test. (B) Bcl‐2 family member siRNAs act by inducing apoptosis in targeted senescent cells (caspase‐3&7 activity assay). Bcl‐2 and Bcl‐xl siRNAs induced apoptosis in HUVECs both on their own, in combinations together, or combined with Bcl‐w siRNA. The combination of Bcl‐2, Bcl‐xl, and Bcl‐w siRNAs, reflecting the targets of N, induced apoptosis to a greater extent in senescent than nonsenescent HUVECs, while the combination of Bcl‐2, Bcl‐xl, and Mcl‐1 siRNAs, reflecting actions of T, did not. Data are means ± SEM of three replicates. *P <* *0.05, ANOVA.

To ascertain why N is senolytic in some but not other cell types as well as why T has less senolytic effect, we analyzed effects of RNA interference against Bcl‐2 family members on viability of senescent HUVECs, IMR90 cells, and primary human preadipocytes (Fig. 4A,B). The ability of the siRNAs to decrease levels of the targeted mRNAs was confirmed by RT–PCR (Fig. S2). As we previously reported, Bcl‐xl siRNA selectively reduced viability of senescent HUVECs but not preadipocytes (Fig. 4A). Unlike in HUVECs (Zhu *et al*., 2015), Bcl‐xl siRNA alone or in combination with Bcl‐2 siRNA did not decrease viability of senescent IMR90 cells. However, the combination of Bcl‐2, Bcl‐xl, and Bcl‐w siRNA was senolytic in IMR90 cells. This combination, which reflects the major targets of N (Vogler, 2014), was also senolytic in HUVECs, but not in preadipocytes, consistent with the sensitivity of senescent HUVECs and IMR90 cells to N and the resistance of preadipocytes. The combination of Bcl‐2, Bcl‐xl, and Mcl‐1 siRNAs, which reflects the main targets of T, was not senolytic in any of these cell types.

### Bcl‐2 family protein signatures

**Figure 5:**
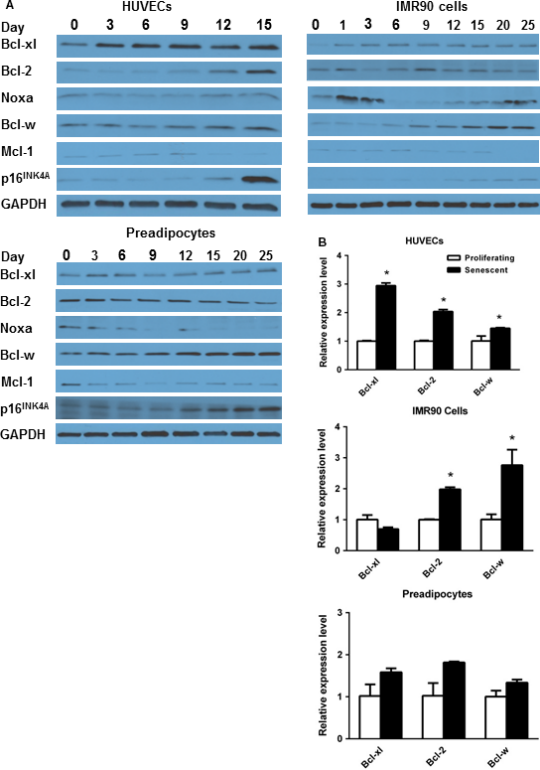
Bcl‐2 family member proteins in senescent HUVECs, IMR90 cells, and human preadipocytes. Bcl‐2 family proteins were followed after 10‐Gy radiation by immunoblot as cells became senescent as indicated by morphology and p16^INK^ ^4A^. (A) representative immunoblots. (B) RT–PCR analyses. Means ± SEM of immunoblots from 3 HUVEC and 3 IMR90 cultures and 3 immunoblots from different subjects. *P <* *0.05, *t*‐test.

Given the varied response of different types of senescent cells to N, we tested whether Bcl‐2 family member protein levels differ between cells that are sensitive or resistant to N. We found patterns of Bcl‐2 family member proteins varied among different cell types as they senesced (Fig. 5; Figs S3–6). In some cases, there were changes shortly after irradiation, consistent with potential acute responses to radiation, while senescence becomes established after days or weeks (Hewitt *et al*., 2012). Bcl‐xl and Bcl‐2 increased in HUVECs, IMR90 cells, and MEFs but not preadipocytes, suggesting that senescent HUVECs, IMR90 cells, and MEFs might depend more on this pro‐survival pathway than N‐resistant preadipocytes. Consistent with this, Bcl‐2 family molecular signatures, including Bcl‐xl, Bcl‐2, Noxa, and Bcl‐w, correctly predict susceptibility to N of 70% of small‐cell lung cancers and 81% of leukemias and lymphomas (Tahir *et al*., 2010; Vo *et al*., 2012). These same Bcl‐2 family molecular signatures are features of senescent HUVECs, IMR90 cells (Fig. 5), and *Ercc1*‐deficient MEFs (Fig. S3), but not preadipocytes (Fig. 5), suggesting that Bcl‐2 signatures may also predict the sensitivity of different types of senescent cells to N, a speculation that merits testing across more senescent cell types.

### Effects of senolytic drug combinations

**Figure 6:**
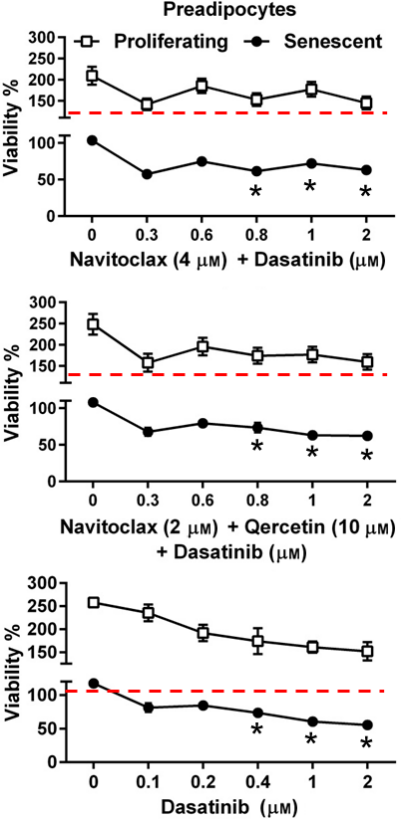
Combining D + N or D + N + Q did not reduce viability (ATPLite) of senescent preadipocytes more than D alone. Proliferating and senescent preadipocytes were exposed to N + Q plus different concentrations of D for 3 days. Means ± SEM of preadipocytes from 4 subjects; *P <* *0.001, ANOVA.

We found no additive effects in preadipocytes from combining D + N or D + N + Q compared to D alone (Fig. 6). We previously reported D + Q did not have a greater senolytic effect in human preadipocytes than D alone (Zhu *et al*., 2015), suggesting that D, but not Bcl‐2 family inhibitors or flavonoids, targets survival pathways needed by senescent preadipocytes. In future studies, it will be important to dissect survival pathways further in senescent preadipocytes, particularly the tyrosine kinase pathways that are targeted by D.

## Discussion

From our hypothesis‐driven, bioinformatics‐based approach, we first predicted that the Bcl‐2 family could be an Achilles heel of senescent cells (Zhu *et al*., 2015). We confirmed this by showing that Bcl‐xl siRNA eliminates senescent but not nonsenescent HUVECs (Zhu *et al*., 2015), a finding that we also confirmed here (Fig. 4). Based on these findings, we tested whether N or T is senolytic. We found that N is senolytic while T is not. Also, similar to D and Q (Zhu *et al*., 2015), N is not a cell type‐independent senolytic agent. It has little, if any, senolytic activity against senescent human primary preadipocytes. Also like D and Q, N is senolytic across species (in certain human and mouse senescent cell types), is effective in cells induced to undergo senescence by different stimuli (radiation and passaging/oxidative stress), and eliminates senescent cells by inducing apoptosis. Furthermore, like D or Q, N is nonspecific, acting on multiple Bcl‐2 family target proteins.

T was less senolytic than N in HUVECs, IMR90 cells, and MEFs, which is likely due to differences in the levels of the targets of N and T. Both N and T target Bcl‐2 and Bcl‐xl, but N also targets Bcl‐w, while T also targets Mcl‐1. Furthermore, unlike HUVECs, which are susceptible to treatment with the Bcl‐xl siRNA alone (Fig. 4; Zhu *et al*., 2015), the Bcl‐xl siRNA on its own was not senolytic in IMR90 cells. However, the combination of Bcl‐2, Bcl‐xl, and Bcl‐w siRNAs, which comprises the constellation of targets of N, was senolytic in IMR90 cells. Thus, for some senescent cell types, candidate senolytic agents that act on multiple pro‐survival targets could be more effective than agents that act solely on a single target, and the particular combination of target proteins that the drugs act on is critical.

The different effects of the related drugs N and T suggest that medicinal chemistry will yield yet more potent senolytics. Consistent with this, we previously found imatinib is not senolytic in preadipocytes despite resembling D structurally, targeting many of the same tyrosine kinases, and having similar clinical indications (Modugno, 2014; Zhu *et al*., 2015). Additionally, N can cause bleeding by affecting platelets and can cause neutropenia, while newer congeners may have less of these effects (Tse *et al*., 2008; Souers *et al*., 2013), further emphasizing a need for medicinal chemical optimization. We caution that people should not take N and physicians should not prescribe it for its senolytic effects unless clinical trials show N is effective and safe for these indications.

While our article was in review, others confirmed and extended our previous finding (Zhu *et al*., 2015) that senolytic agents have a wide range of potentially beneficial effects for a number of senescence‐related indications *in vivo* and noted that N is senolytic *in vitro* and *in vivo* (Chang *et al*., 2015). As we first demonstrated for the senolytics D + Q (Zhu *et al*., 2015), N also was found to be radioprotective *in vivo* (Chang *et al*., 2015). In that report, some human‐origin cell lines (as opposed to primary cells) were used, with N having senolytic activity in each of the lines selected. Based on this observation, it was suggested that Bcl‐2 family inhibitors have a broad spectrum of activity, being senolytic in a cell type‐independent manner, unlike D or Q. Here we observed that the senescent cell types that N can target are in fact limited. N is not senolytic against senescent human primary preadipocytes, unlike the tyrosine kinase inhibitor, D. This is consistent with our previous finding that while Bcl‐xl siRNA reduces viability of senescent HUVECs, it is not senolytic in primary preadipocytes (Zhu *et al*., 2015). Senescent preadipocytes are potentially important and arguably among the most abundant types of senescent cell in humans (Tchkonia *et al*., 2010).

We also observed that a combination of Bcl‐2 family member siRNAs interferes with viability of IMR90 cells and that Bcl‐xl siRNA alone is not effective in this particular senescent cell type, as also noted by others (Chang *et al*., 2015). This is unlike senescent HUVECs, which are susceptible to Bcl‐xl siRNA alone (Fig. 4; Zhu *et al*., 2015). Thus, responses of various senescent cell types vary to interventions targeting different members of the Bcl‐2 family, reinforcing the value of developing agents that have multiple targets, like D, Q, or N.

Senolytics could be valuable in treating disorders related to senescent cell accumulation, for example, atherosclerosis, chronic obstructive lung disease, idiopathic pulmonary fibrosis, osteoarthritis, diabetes, kidney dysfunction, dementias, and neurodegenerative diseases (Tchkonia *et al*., 2013; Zhu *et al*., 2014; Palmer *et al*., 2015). It appears that the senolytics described so far, including D, Q, and now N, are limited in the senescent cell types they can target, underscoring the value of testing each cell type involved in particular diseases of interest as part of the senolytic drug development process. We speculate that it may be possible to base selection of senolytic drugs for a particular disease indication on the molecular profiles of the types of senescent cells that underlie that disease. Furthermore, combination treatments for certain indications involving multiple senescent cell types may be optimal in some cases. Overall, our findings support the feasibility of using our hypothesis‐driven, bioinformatics‐based strategy (Zhu *et al*., 2015) to develop more, perhaps better senolytic agents than D, Q, or N. Furthermore, it appears feasible to develop senolytic agents that target senescent cells of a particular type, in a particular tissue, or for a particular indication.

## Experimental procedures

### Preadipocyte isolation and culture

Primary human preadipocytes were isolated from healthy, lean kidney transplant donors. The protocol was approved by the Mayo Clinic Foundation Institutional Review Board for Human Research. Detailed descriptions of our preadipocyte, HUVEC, IMR90, and MEF culture methods are in Data S1 (Supporting information) and publications (Tchkonia *et al*., 2007; Wang *et al*., 2012).

### Induction of cellular senescence

HUVECs, IMR90 cells, or preadipocytes at passage 4 were radiated at 10 Gy to induce senescence or were sham‐radiated. Preadipocytes were senescent by 20 days after radiation, IMR90 cells after 20 days, and HUVECs after 14 days, exhibiting SA‐βGal positivity and SASP factor expression by ELISA (IL‐6, MCP‐1). MEFs were induced to become senescent by passaging under high oxygen (20%) conditions as in (Zhu *et al*., 2015b).

### Senescence‐associated β‐galactosidase activity

Cellular senescence‐associated β‐galactosidase (SA‐βGal) activity was assayed as previously described (Stout *et al*., 2014) using 8–10 images taken of random fields from each sample by fluorescence microscopy.

### RNA methods

siRNAs are described in Table S1 (Supporting information). Cells were transduced with siRNA using Lipofectamine and harvested 24 h after transduction. RT–PCR methods are in our publications (Cartwright *et al*., 2010). TATA‐binding protein (TBP) was used as internal control.

### Other methods

Cell viability, apoptosis, and immunoblotting methods are described in Data S1 (Supporting information) and one of our publications (Zhu *et al*., 2015).

## Author contributions

YZ performed experiments on human cells; HFS and YYL on MEFs. JLK, YZ, TT, HFS, HMD, CBG, JDW, MBS, LN, and PR contributed to experimental design, data analysis, and manuscript preparation.

## Funding

This work was supported by NIH grants AG13925 (JLK), DK50456 (JLK: Adipocyte Subcore), AG043376 (PDR&LJN), the Noaber, Ellison, the Ted Nash and Glenn Foundations, the American Federation for Aging Research, the Connor Group, and the Natural Science Foundation of China (HMD: grant 81572948).

## Conflict of interest

YZ, TT, TP, NG, LJN, PDR, and JLK have potential financial interest in this research. The research has been reviewed by the Mayo Clinic Conflict of Interest Review Board and was conducted in compliance with Mayo Clinic and the Scripps Research Institute (TSRI) conflict of interest policies.

## Supporting information


**Fig. S1** Representative TUNEL assay photographs in senescent and nonsenescent HUVECs and IMR90 cells.
**Fig. S2** Confirmation of siRNA‐induced decreases in target mRNAs by RT–PCR.
**Fig. S3** Bcl‐2 family member proteins in senescent vs. nonsenescent *Ercc1*‐deficient MEFs.
**Fig. S4–6** Densitometric analyses of Bcl‐2 family member proteins in senescent vs. nonsenescent in HUVECs, IMR90 cells, and primary human preadipocytes, respectively.

**Table S1** siRNAs and primers.

**Data S1** Experimental procedures.
