# Supplementary material for: Identification of a novel senolytic agent, navitoclax, targeting the Bcl‐2 family of anti‐apoptotic factors
Source: Aging Cell. 2016 Mar 18;15(3):428–35. doi: 10.1111/acel.12445 (PMC4854923; doi:10.1111/acel.12445)

Supplemental Figure 1

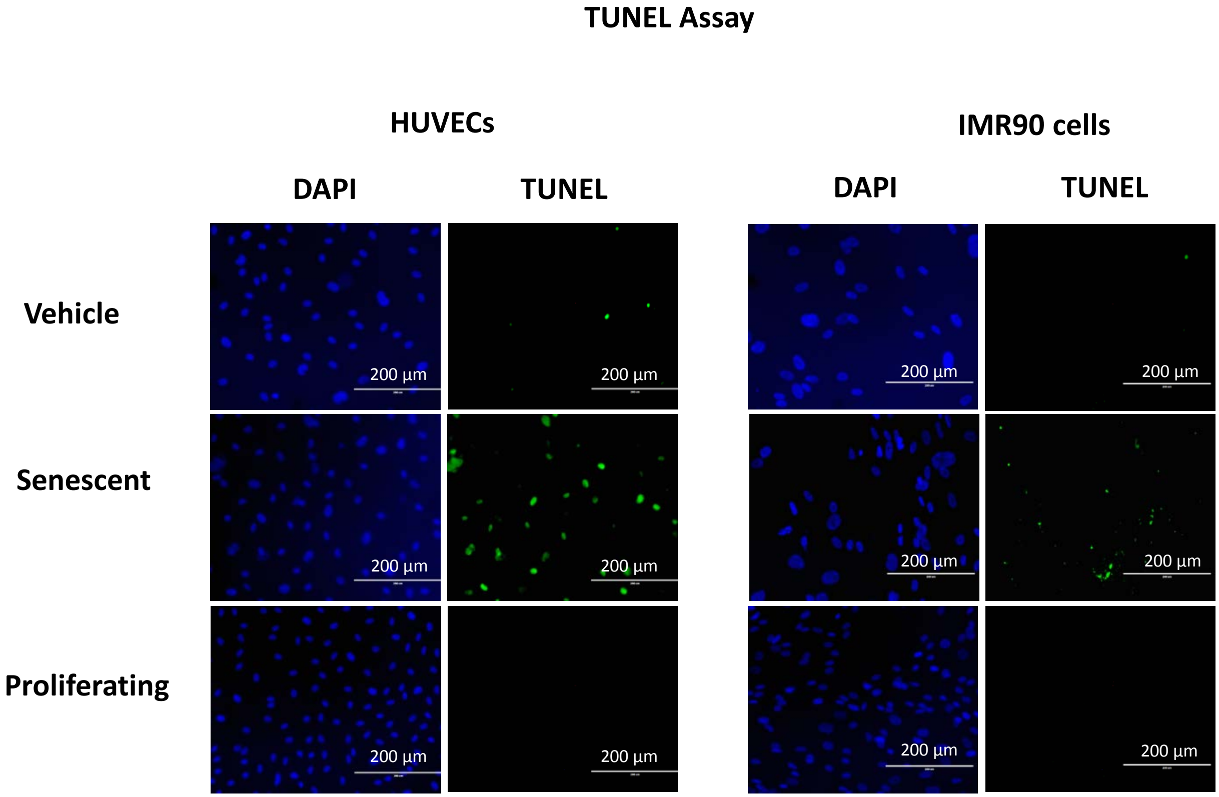

Supplemental Figure 2

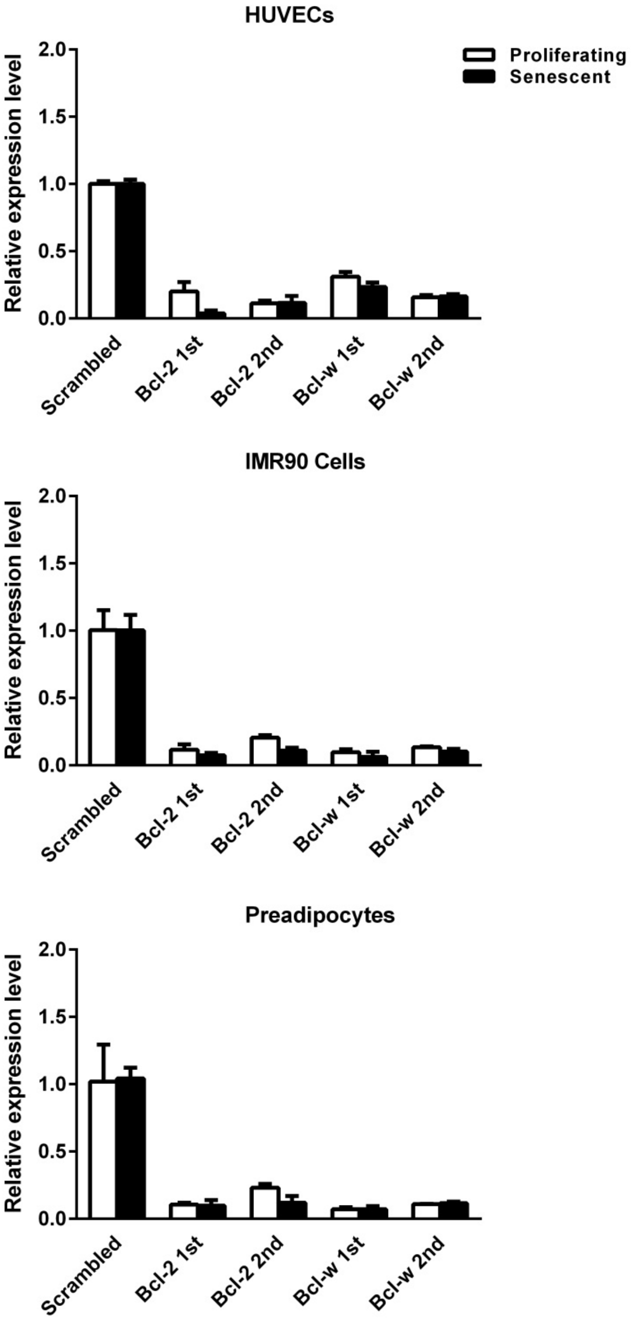

Supplemental Figure 3

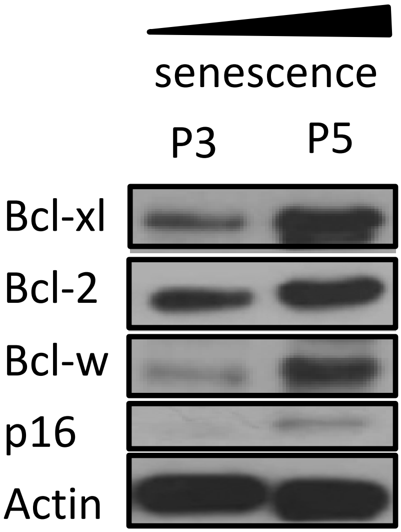

Supplemental Figure 4

HUVECs

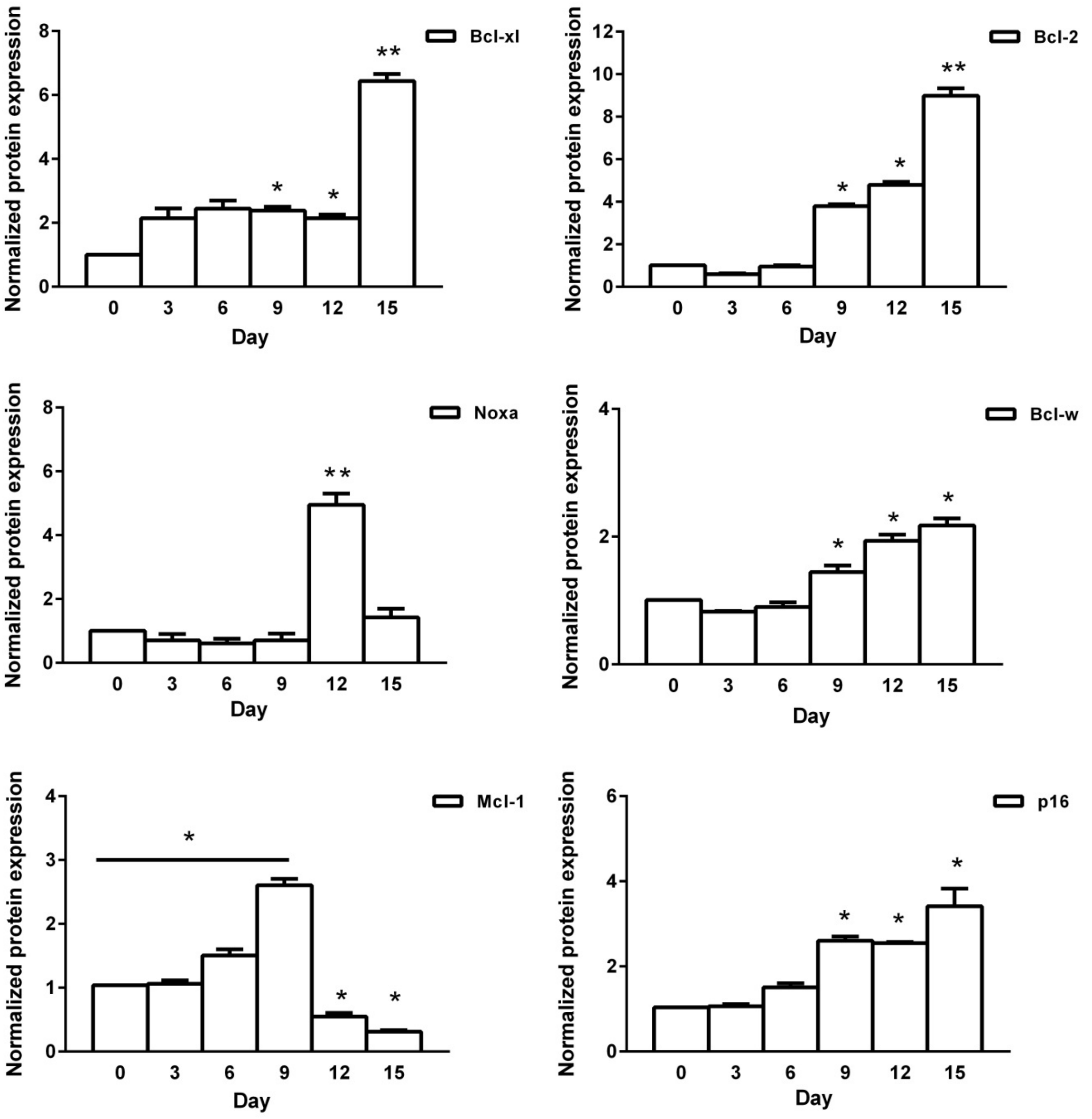

Supplemental Figure 5

IMR90 Cells

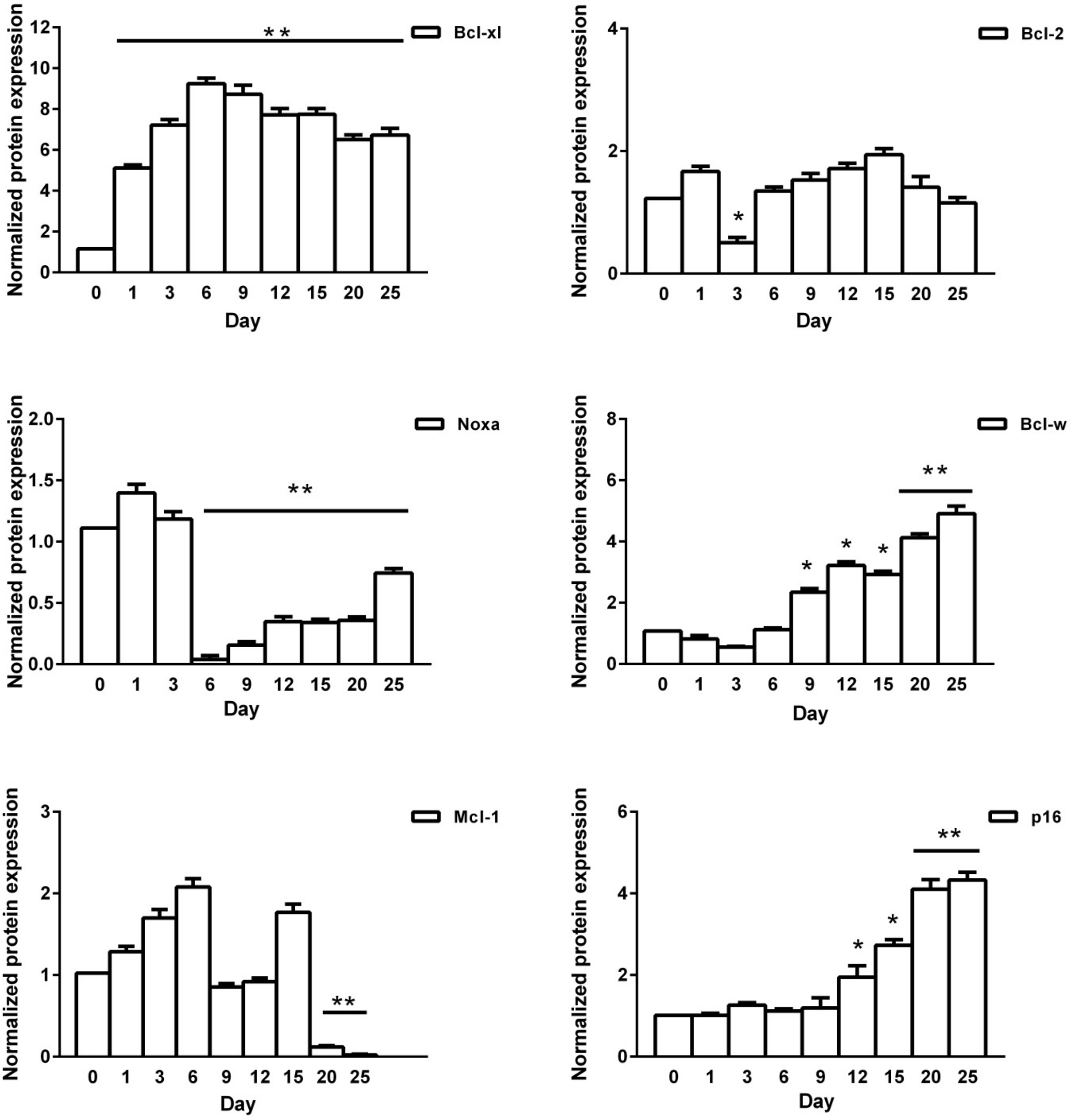

Supplemental Figure 6

Preadipocytes

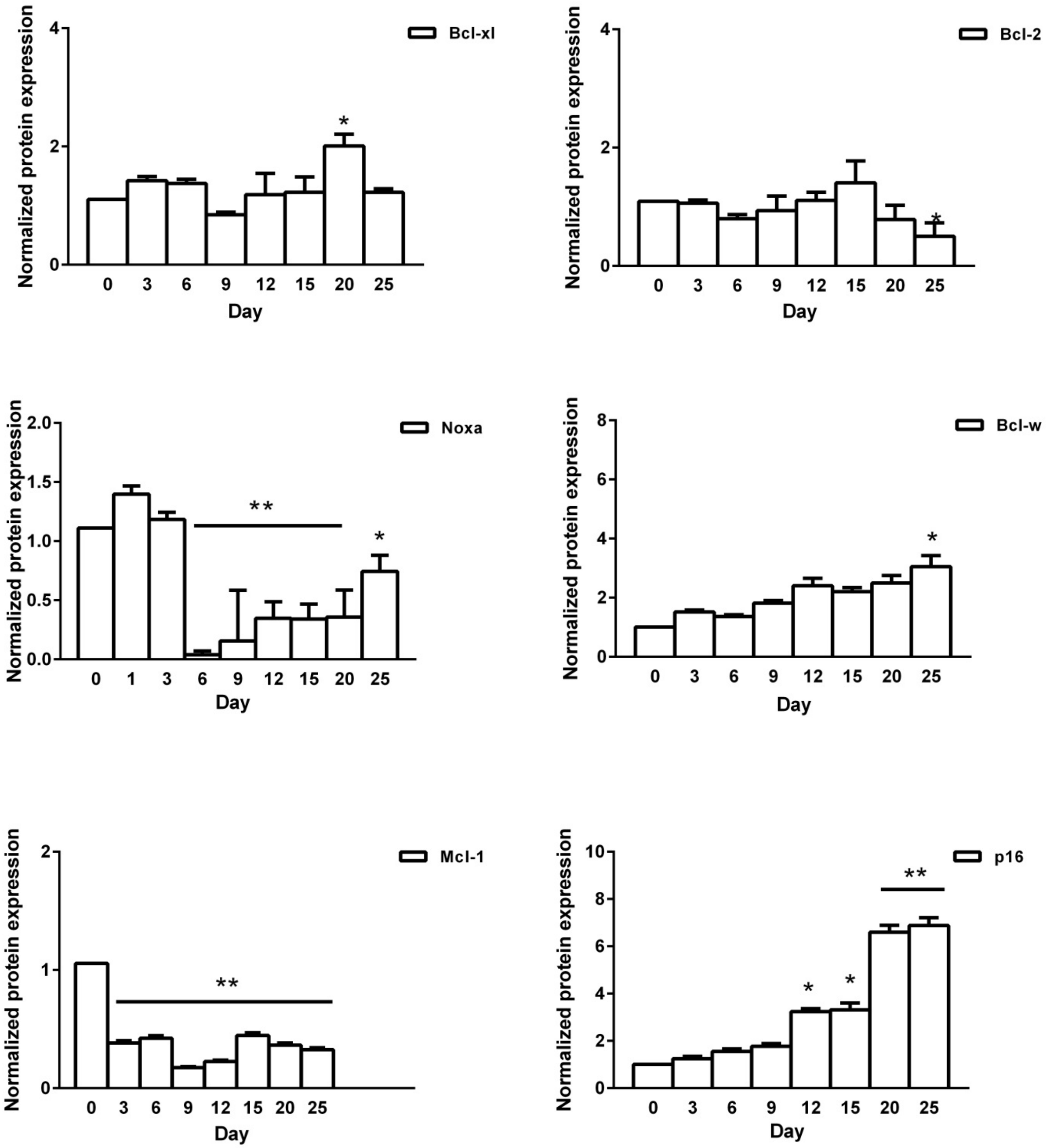

Supplement: Supplementary file 1 — Fig. S1 Representative TUNEL assay photographs in senescent and nonsenescent HUVECs and IMR90 cells. Fig. S2 Confirmation of siRNA‐induced decreases in target mRNAs by RT–PCR. Fig. S3 Bcl‐2 family member proteins in senescent vs. nonsenescent Ercc1‐deficient MEFs. Fig. S4–6 Densitometric analyses of Bcl‐2 family member proteins in senescent vs. nonsenescent in HUVECs, IMR90 cells, and primary human preadipocytes, respectively. [file ACEL-15-428-s001.pdf]
