# Supplementary material for: Identification of a novel senolytic agent, navitoclax, targeting the Bcl‐2 family of anti‐apoptotic factors
Source: Aging Cell. 2016 Mar 18;15(3):428–35. doi: 10.1111/acel.12445 (PMC4854923; doi:10.1111/acel.12445)
Supplement: Supplementary file 2 — Table S1 siRNAs and primers. [file ACEL-15-428-s002.docx]

**Supplemental Table 1: List of Human siRNA and TagMan Primer Probe Assays**

| siRNA | Cat. # |
| --- | --- |
| BCL2 #1 | 289161 |
| BCL2 #2 | 214532 |
| BCL2L2 #1 | 120365 |
| BCL2L2 #2 | 5027 |
| BCL2L1 | 120716 |
| MCL1 | 120642 |
| Primers | Cat. # |
| BCL2 | Hs00608023_m1 |
| BCL2L2 | Hs00187848_m1 |
| BCL2L1 | Hs00236329_m1 |
| MCL1 | Hs01050896_m1 |
