# Supplementary material for: Identification of a novel senolytic agent, navitoclax, targeting the Bcl‐2 family of anti‐apoptotic factors
Source: Aging Cell. 2016 Mar 18;15(3):428–35. doi: 10.1111/acel.12445 (PMC4854923; doi:10.1111/acel.12445)
Supplement: Supplementary file 3 — Data S1 Experimental procedures. [file ACEL-15-428-s003.docx]

**Data S1. Materials and Methods**

*Preadipocyte isolation and culture*

Adipose tissue for preadipocyte isolation was obtained during intra-abdominal surgery from 4 healthy kidney transplant donors (male; age 39.3±2.1 [mean±SEM] years; BMI, 24±1.7) who had given informed consent. The protocol was approved by the Mayo Clinic Foundation Institutional Review Board for Human Research. Detailed methods for preadipocyte culture were previously published ([Caserta *et al.* 2001](#_ENREF_1); [Karagiannides *et al.* 2001](#_ENREF_2); [Tchkonia *et al.* 2002](#_ENREF_4); [Tchkonia *et al.* 2005](#_ENREF_7); [Karagiannides *et al.* 2006](#_ENREF_3); [Tchkonia *et al.* 2006](#_ENREF_5); [Tchkonia *et al.* 2007](#_ENREF_6); [Zhu *et al.* 2015](#_ENREF_10)). Note that preadipocytes are also known as fat cell progenitors or adipose-derived stem cells (for a discussion of nomenclature, see ([Tchkonia *et al.* 2013](#_ENREF_8))). These cells are in the stromal vascular fraction of adipose tissue collagenase digests. Cells used had been passaged for 4 population doublings.

*Human Umbilical Vein Endothelial Cell (HUVEC) culture and reagents*

Human umbilical vein endothelial cells (HUVECs) were purchased from Lonza (Walkersville, MD) and grown in Clonetics Endothelial Cell Growth Medium-2 (EGM-2; Lonza) following the manufacturer’s protocol ([Wang *et al.* 2012](#_ENREF_9)).

*Human lung fibroblast (IMR90) cell culture and reagents*

IMR90 cells (passage 10) were purchased from ATCC (ATCC; Manassas, VA) and grown in Eagle's Minimum Essential Medium (ATCC-30-2003; ATCC) following the manufacturer’s protocol.

*Induction of cellular senescence*

Preadipocytes, IMR90 cells, or HUVECs were irradiated in a RS2000 X-Ray Irradiator (RAD Source Technologies; Suwanee, GA) at 10 Gy to induce senescence or were sham-radiated to grow cultures of proliferating cells. Preadipocytes were senescent by 20 days after irradiation, IMR90 cells were senescent by 20 days, and HUVECs by 14 days, exhibiting abnormal morphology, increased SA-βGal staining, and increased p16^INK4A^ expression as determined by immunoblotting ([Zhu *et al.* 2015](#_ENREF_10)).

*Chemicals*

Navitoclax and TW-37 were purchased from Selleckchem (cat #S1001, cat#S1121, respectively; Houston, TX). Quercetin was purchased from Sigma (cat#1592409; St. Louis, MO). Dasatinib was purchased from LC Laboratories (cat# D-3307; Woburn, MA).

*Mouse Embryonic Fibroblast (MEF) isolation*

The *Ercc1*^-/-^ or wild-type MEFs were isolated from pregnant female mice at ~day 13 post-coitus and cultured in a 1:1 mixture of Dulbecco’s modified Eagle’s medium and Ham’s F10 with 10% fetal bovine serum, 1x nonessential amino acids, penicillin, and streptomycin and incubated at 3% O_2_ initially, followed by a shift to 20% for 5 passages to induce senescence ([Zhu *et al.* 2015](#_ENREF_10)). Wild type cells were kept at 3% O_2_.

*Analysis of effects of drugs on senescent MEFs*

5x10^3^ MEFs at passage 5 in 20% O_2_ were seeded per well in 96-well plates 6 h prior to drug treatment. Following the addition of drugs, the MEFs were incubated for 48 h at 20% O_2_. For fluorescence analysis of cellular SA-ßGal activity, the cells were washed once with PBS, C_12_FDG (10 µM) was added to the culture medium and the cells were incubated for 1.5–2 h. Approximately 50% of the cells were SA-ßGal positive. Ten min prior to analysis, DNA intercalating Hoechst dye (2 µg/ml), was added to the cultures. An InCell Analyzer 6000, a laser-based line scanning confocal imager with large field-of-view sCMOS camera detection technology, was used for the quantitative detection of cell number (Hoechst staining) and the number of C_12_FDG positive, senescent cells. Relative cell senescence was determined by comparing the percent of senescent cells in each cell culture to vehicle-treated cells.

*Cell viability and apoptosis assay in preadipocytes, IMR90 cells, and HUVECs*

Cell viability after drug treatment was measured by ATPLite Kit (cat# 6016941; PerkinElmer; Waltham, MA). The assay was performed following the manufacturer’s instructions. Luminescence was read using a multi-scan plate reader (Fisher; Waltham, MA). For apoptosis, a caspase 3&7 activity assay was performed in 96-well plates using a Caspase-Glo® or APO-ONE 3/7 Assay Systems kits (Promega; Madison, WI). To confirm apoptosis, DeadEnd™ Fluorometric TUNEL assays (Promega) were performed. Cells were cultured on 8-well tissue culture chamber slides (BD Falcon; Bedford, MA) and then treated with either DMSO (vehicle only) or drugs for 8-24 h. The assays were performed following the manufacturer’s instructions.

*Immunoblotting*

25µg total protein isolated from cultured cells was loaded per lane for immunodetection*.* Antibodies used in the study were rabbit anti-Bcl-xl (1:1000, #2764; Cell Signaling; Danvers, MA), mouse anti-Bcl-2 (1:500, #M0887; Dako; Carpinteria, CA), rabbit anti-Mcl-1 (1:1000, #5453; Cell Signaling), rabbit anti-Bcl-w (1:1000, #2724; Cell Signaling), rabbit anti-Noxa (1:1000, #14766; Cell Signaling), rabbit anti-p16^INK4A^ (1:1000, #10883-1-AP; Proteintech; Chicago, IL), and rabbit anti-GADPH (1:1000, #5174; Cell Signaling) as a loading control. The secondary antibodies included anti-rabbit or anti-mouse IgG obtained from Santa Cruz (Dallas, TX).

**References**

Caserta F, Tchkonia T, Civelek V, Prentki M, Brown NF, McGarry JD, Forse RA, Corkey BE, Hamilton JA, Kirkland JL (2001). Fat depot origin affects fatty acid handling in cultured rat and human preadipocytes. *Am. J. Physiol.* **280**, E238-E247.

Karagiannides I, Tchkonia T, Dobson DE, Steppan CM, Cummins P, Chan G, Salvatori K, Hadzopoulou-Cladaras M, Kirkland JL (2001). Altered expression of C/EBP family members results in decreased adipogenesis with aging. *Am. J. Physiol.* **280**, R1772-R1780.

Karagiannides I, Thomou T, Tchkonia T, Pirtskhalava T, Kypreos KE, Cartwright A, Dalagiorgou G, Lash TL, Farmer SR, Timchenko NA, Kirkland JL (2006). Increased CUG triplet repeat binding protein-1 predisposes to impaired adipogenesis with aging. *J. Biol. Chem.* **281**, 23025-23033.

Tchkonia T, Giorgadze N, Pirtskhalava T, Tchoukalova Y, Karagiannides I, Forse RA, DePonte M, Stevenson M, Guo W, Han J, Waloga G, Lash TL, Jensen MD, Kirkland JL (2002). Fat depot origin affects adipogenesis in primary cultured and cloned human preadipocytes. *Am. J. Physiol.* **282**, R1286-R1296.

Tchkonia T, Giorgadze N, Pirtskhalava T, Thomou T, DePonte M, Koo A, Forse RA, Chinnappan D, Carmen Martin-Ruiz C, von Zglinicki T, Kirkland JL (2006). Fat depot-specific characteristics are retained in strains derived from single human preadipocytes. *Diabetes*. **55**, 2571-2578.

Tchkonia T, Lenburg M, Thomou T, Giorgadze N, Frampton G, Pirtskhalava T, Cartwright A, Cartwright M, Flanagan J, Karagiannides I, Gerry N, Forse RA, Tchoukalova Y, Jensen MD, Pothoulakis C, Kirkland JL (2007). Identification of depot-specific human fat cell progenitors through distinct expression profiles and developmental gene patterns. *Am. J. Physiol.* **292**, E298-E307.

Tchkonia T, Tchoukalova YD, Giorgadze N, Pirtskhalava T, Karagiannides I, Forse RA, Koo A, Stevenson M, Chinnappan D, Cartwright A, Jensen MD, Kirkland JL (2005). Abundance of two human preadipocyte subtypes with distinct capacities for replication, adipogenesis, and apoptosis varies among fat depots. *Am. J. Physiol.* **288**, E267-E277.

Tchkonia T, Thomou T, Zhu Y, Karagiannides I, Pothoulakis C, Jensen MD, Kirkland JL (2013). Mechanisms and metabolic implications of regional differences among fat depots. *Cell Metab*.

Wang S, Xu M, Li F, Wang X, Bower KA, Frank JA, Lu Y, Chen G, Zhang Z, Ke Z, Shi X, Luo J (2012). Ethanol promotes mammary tumor growth and angiogenesis: the involvement of chemoattractant factor MCP-1. *Breast Cancer Res Treat*. **133**, 1037-1048.

Zhu Y, Tchkonia T, Pirtskhalava T, Gower A, Ding H, Giorgadze N, Palmer AK, Ikeno Y, Borden G, Lenburg M, O'Hara SP, LaRusso NF, Miller JD, Roos CM, Verzosa GC, LeBrasseur NK, Wren JD, Farr JN, Khosla S, Stout MB, McGowan SJ, Fuhrmann-Stroissnigg H, Gurkar AU, Zhao J, Colangelo D, Dorronsoro A, Ling YY, Barghouthy AS, Navarro DC, Sano T, Robbins PD, Niedernhofer LJ, Kirkland JL (2015). The Achilles' heel of senescent cells: From transcriptome to senolytic drugs. *Aging Cell*.
