# Supplementary material for: Identification of a novel senolytic agent, navitoclax, targeting the Bcl‐2 family of anti‐apoptotic factors
Source: Aging Cell. 2016 Mar 18;15(3):428–35. doi: 10.1111/acel.12445 (PMC4854923; doi:10.1111/acel.12445)
Supplement: Supplementary file 4 [file ACEL-15-428-s004.docx]

**Supplemental Fig. S1** Like dasatinib and quercetin ([Zhu *et al.* 2015b](#_ENREF_31)), navitoclax causes senescent cell death by apoptosis. Navitoclax causes apoptosis of senescent HUVECs and IMR90 cells. Terminal deoxynucleotidyl transferase dUTP nick end labeling (TUNEL) with fluorescent green dye shows apoptotic cells in cultures of irradiated, senescent cells.

**Supplemental Fig. S2** Confirmation of siRNA-induced decreases in target mRNAs by RT-PCR. Extent of mRNA knock-down by siRNA’s were equivalent in senescent (solid bars) to non-senescent (open bars) HUVECs, IMR90 cells, and primary human preadipocytes. 1^st^ refers to one siRNA and 2^nd^ refers to a second confirmatory siRNA. Forty-eight hours after cells were transfected with siRNA’s cells were harvested and mRNA was quantified by RT-PCR. For each cell type, N=3 independent replicates. Means±SEM are shown.

**Supplemental Fig. S3** Bcl-2 family member proteins in senescent *vs*. non-senescent *Ercc1*-deficient MEFs. Bcl-2 family members and p16 were detected by immunoblotting. Passage 3 (P3) and 5 (P5) cultures were analyzed. β- actin (Actin) is shown as a loading control. Representative of 3 experiments.

**Supplemental Fig. S4** Densitometric analyses of immunoblots in HUVECs from Fig. 5. “Normalized” refers to the ratio to control, non-senescent cells. Means±SEM of 3 replicates are shown. **P*<0.05; ***P*<0.01; *t* test.

**Supplemental Fig. S5** Densitometric analyses of immunoblots in IMR90 cells from Fig. 5. “Normalized” refers to the ratio to control, non-senescent cells. Means±SEM of 3 replicates are shown. **P*<0.05; ***P*<0.01; *t* test.

**Supplemental Fig. S6** Densitometric analyses of immunoblots in human preadipocytes from Fig. 5. “Normalized” refers to the ratio to control, non-senescent cells. Means±SEM of immunoblots from 3 subjects are shown. **P*<0.05; ***P*<0.01; *t* test.

**Table S1** siRNAs and primers. siRNAs were from Life Technologies (Grand Island, NY) and PCR primers were from Applied Biosystems (Foster City, CA).
